# Isolation of an Angiotensin I-Converting Enzyme Inhibitory Protein with Antihypertensive Effect in Spontaneously Hypertensive Rats from the Edible Wild Mushroom *Leucopaxillus tricolor*

**DOI:** 10.3390/molecules200610141

**Published:** 2015-06-01

**Authors:** Xueran Geng, Guoting Tian, Weiwei Zhang, Yongchang Zhao, Liyan Zhao, Mansok Ryu, Hexiang Wang, Tzi Bun Ng

**Affiliations:** 1 State Key Laboratory for Agrobiotechnology and Department of Microbiology, China Agricultural University, Beijing 100193, China; E-Mails: gengxueran2007@163.com (X.G.); zhangweiweicau@gmail.com (W.Z.); 13161825783@163.com (M.R.); 2 Institute of Biotechnology and Germplasmic Resource, Yunnan Academy of Agricultural Science, Kunming 650223, China; E-Mails:tiangt@aliyun.com (G.T.); yaasmushroom@aliyun.com (Y.Z.); 3 College of Food Science and Technology, Nanjing Agricultural University, Weigang, Nanjing 210095, China; E-Mail: zhlychen@njau.edu.cn; 4 School of Biomedical Sciences, Faculty of Medicine, The Chinese University of Hong Kong, Shatin, New Territories, Hong Kong, China

**Keywords:** *Leucopaxillus tricolor*, ACE inhibitory protein, inhibitory pattern, spontaneously hypertensive rat

## Abstract

An 86-kDa homodimeric angiotensin I-converting enzyme (ACE) inhibitory protein designated as LTP was isolated from fruit bodies of the mushroom *Leucopaxillus tricolor*. The isolation procedure involved ultrafiltration through a membrane with a molecular weight cutoff of 10-kDa, ion exchange chromatography on Q-Sepharose, and finally fast protein liquid chromatography-gel filtration on Superdex 75. LTP exhibited an IC_50_ value of 1.64 mg∙mL^−1^ for its ACE inhibitory activity. The unique N-terminal amino acid sequence of LTP was disclosed by Edman degradation to be DGPTMHRQAVADFKQ. In addition, seven internal sequences of LTP were elucidated by liquid chromatography-tandem mass spectrometry (LC-MS/MS) analysis. Results of the Lineweaver-Burk plot suggested that LTP competitively inhibited ACE. Both LTP and the water extract of *L. tricolor* exhibited a clear antihypertensive effect on spontaneously hypertensive rats.

## 1. Introduction

According to the World Health Organization (WHO) update in May 2014, cardiovascular disease ranks top among the 10 leading causes of death worldwide. One of the major risk factors for cardiovascular disease is hypertension. It has been estimated that hypertension currently kills nine million people every year [1,2].

Arterial blood pressure is regulated by various factors, including the renin-angiotensin-aldosterone system (RAS). Angiotensin converting enzyme (ACE, EC. 3.4.15.1) is a Zn-metallopeptidase which plays a key role in regulating blood pressure in the RAS. The primary function of ACE is to catalyze the conversion of Ang-I (a decapeptide) to Ang-II (an octapeptide). Ang-II, a potent vasoconstrictor, interacts with the Ang-II type 1 receptor (AT1) and stimulate the secretion of aldosterone, which enhances sodium and water re-absorption in the nephron and therefore elevates blood pressure by increasing the intravascular fluid volume [3,4,5]. The use of ACE inhibitors has proven to be an effective strategy in the prevention and treatment of hypertension-related diseases, mainly by bringing about a significant alleviation of hypertension, as well as by affording end-organ protection [6]. Since the first discovery in snake venom [7], a diversity of ACE inhibitors including captopril, enalapril, alacepril, lisinopril, perindopril, fosinopril, ramipril, trandolapril, and zofenopril has been synthesized and used clinically as antihypertensive drugs [8,9]. Following the administration of synthetic ACE inhibitors, side effects including coughing, taste disturbances and allergic reactions have been reported. Thus it would be desirable to ascertain and use safe, novel, and relatively inexpensive ACE inhibitors from natural sources.

Many reports are available on bioactive natural constituents from food sources, e.g., grains, fruits, vegetables, nuts, sea foods, dairy products, meats and foods of microbial origin that have potential use in the prevention and treatment of hypertension [10]. Mushrooms are popular not only because of their delicious taste, but also due to their proven nutritional and medicinal effects. Studies on isolation of ACE inhibitors from wild mushrooms are currently lacking in the literature. Recently, researchers have reported that an aqueous extract of *Pleurotus ostreatus* [11] and an ACE inhibitory peptide from *Agaricus bisporus* [12] have the potential to inhibit ACE and reduce blood pressure. ACE inhibitory activities of wild mushrooms in Nepal have been tested by Bang and his group [13]. *Leucopaxillus tricolor* is an edible mushroom which is popular among local Chinese people due to its medicinal value and wide distribution in grasslands. To the best of our knowledge, there are no publications on the biological activity of this mushroom.

Hence, the objectives of this study were to examine the ACE inhibitory activities of fruit body extracts of different mushroom species, purify any ACE inhibitors from the mushrooms, and to subject the crude mushroom extracts and purified ACE inhibitors to *in vivo* tests of antihypertensive activity using an animal model of hypertension. The results of this study disclosed the therapeutic potential of the wild mushroom *L. tricolor* and added a new *L. tricolor* ACE inhibitor to the existing pool of ACE inhibitors.

## 2. Results and Discussion

### 2.1. ACE Inhibitory Activities of Fruit Body Extracts of Different Mushroom Species

Extracts from the fruit bodies of 10 wild mushrooms were prepared and their ACE inhibitory activities were tested. As shown in Table 1, the percentages of ACE inhibition achieved were spread over a wide range from 29.2%–95%. The highest ACE inhibitory activity (up to 95% inhibition) was displayed by *L. tricolor*, followed by *Grifola frondosa* and *Boletus bicolor*. The lowest ACE inhibitory activity (30.8% and 29.2% inhibition, respectively) was demonstrated by *Oudemansiella radicata* and *Gloeostereum incarnatum*. In this study, the water extract of *G. frondosa* inhibited ACE activity by 77.2%, whereas 58.7% inhibition was reported by Choi *et al*. [14]. Different strains, different extraction methods, and detection methods employed by different investigators may have contributed to a large variation in the ACE inhibitory activity observed. For instance, the extract of *P. sajor-caju* inhibited the activity of ACE by 85.2% as reported by Lau *et al*. [15]. On the other hand, in the study of Lee *et al.* [16], the water extract of this mushroom inhibited ACE activity by 38.7%. The most potent ACE inhibitory activity was pursued further in the current investigation.

**Table 1:** ACE inhibitory activities of water extracts of various mushrooms ^a^.

Mushroom Species	ACE Inhibitory Activity (%) of Water Extract
*Leucopaxillus tricolor*	95.0% ± 0.4
*Grifola frondosa*	77.2% ± 0.5
*Boletus bicolor*	61.3% ± 0.1
*Tuber micheli*	56.5% ± 0.1
*Russula aeruginea*	53.1% ± 0.7
*Boletus edulis*	47.2% ± 0.2
*Morchella vulgaris*	43.3% ± 0.2
*Ramaria botrytoides*	37.8% ± 0.0
*Oudemansiella radicata*	30.8% ± 0.1
*Gloeostereum incarnatum*	29.2% ± 0.9

^a^ The ratio of mushroom to distilled water (weight to volume) used for preparing the extracts was 1:2. Values were means ± S.D. of three determinations.

### 2.2. Purification of Potential ACE Inhibitor

**Figure 1:**
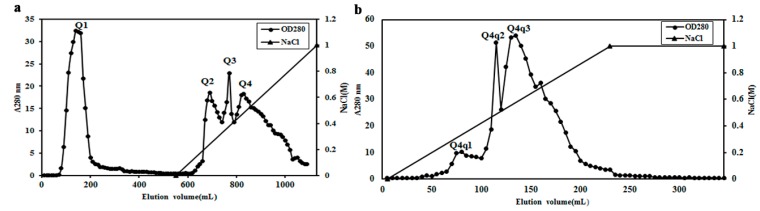
Profiles of purification obtained by ion-exchange chromatography on (**a**) the first Q-Sepharose column (3 cm × 25 cm) and (**b**) the second Q-Sepharose column (3 cm × 15 cm).

**Figure 2:**
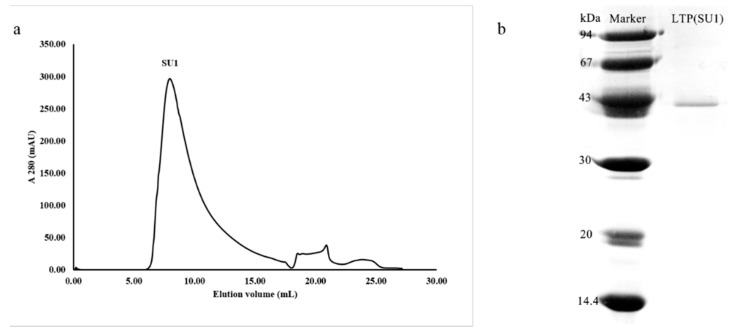
(**a**) FPLC-gel filtration on Superdex 75 10/300 GL column and (**b**) SDS-PAGE of fraction SU1. ^a^ Eluent: 10 mM Tris-HCl buffer (pH 7.5). Fraction size: 0.8 mL. Flow rate: 0.5 mL∙min^−1^. Fraction SU1 represents purified ACE inhibitory protein; ^b^ SDS-PAGE of fraction SUI.

*L. tricolor* ACE inhibitor purification was guided by bioassay. The purification procedure entailed extraction with distilled water, ultrafiltration through a 10-kDa MWCO membrane, followed by ion-exchange chromatography on Q-Sepharose, and finally FPLC on a Superdex 75 column. After the water extract of *L. tricolor* had been ultrafiltered with a 10-kDa cut-off filter, the cake produced 86.3% inhibition while the filtrate brought about only 41.4% inhibition of the ACE activity and thus the cake was employed for further isolation. After adjustment of the pH, the solution of the cake was loaded on a Q-Sepharose column and eluted with a linear gradient of 0–1 M NaCl in 10 mM Tris-HCl buffer (pH 7.5). Four fractions, Q1, Q2, Q3 and Q4, were obtained (Figure 1a). The bulk of ACE inhibitory activity resided in fraction Q4. When fraction Q4 was subjected to chromatography on another Q-Sepharose column (pH 7.2), the ACE inhibitory activity was located in fraction Q4q2 (Figure 1b). This fraction was further purified on Superdex 75, yielding essentially a single peak (Figure 2a). The first and highest peak (SU1) was the purified ACE inhibitor. According to the elution volume of the peak in FPLC-gel filtration, the molecular mass of the inhibitor named LTP was determined to be about 86 kDa.

The unadsorbed fraction Q1 was eluted with 10 mM Tris-HCl buffer (pH 7.5). The adsorbed fraction was eluted from the column using a linear (0–1 M) NaCl gradient in Tris-HCl buffer (pH 7.5) as indicated by the slanting line across the right half of the chromatogram. Q4 was the active fraction. (b) Fraction Q4 was loaded on a Q-Sepharose column (3 cm × 15 cm). The line with triangular symbols indicates elution of adsorbed proteins with a 0–1 M NaCl gradient in 10 mM Tris-HCl buffer (pH 7.2). ACE inhibitory activity was detected only in fraction Q4q2.

The documented bioactive substances for reducing blood pressure include various peptides, phenolic compounds, proteins, polysaccharides, saponins, sterols, pigments, fiber, vitamins C and E, as well as minerals [10]. A tuber storage protein from yam called dioscorin (32 kDa) purified by DE-52 ion-exchange chromatography, exhibited ACE inhibitory activity [17]. The protein thioredoxin h2 (14 kDa) and a trypsin inhibitor, both from sweet potato storage root, were ACE inhibitors, with IC_50_ values of 151.8 mg∙mL^−1^ and 187.96 mg∙mL^−1^, respectively [18,19]. Mycelia of the edible mushroom *Ganoderma* *lucidum* contained several antihypertensive-related proteins, including cystathionine beta synthase-like protein, DEAD/DEAH box helicase-like protein, paxillin-like protein, and alpha/beta hydrolase-like protein, thus *G. lucidum* has potential in lowering blood pressure [20]. All aforementioned peptides and proteins are exogenous inhibitors. Recently, by ultrafiltering serum, an endogenous ACE inhibitory protein with a molecular weight of about 50–100 kDa was discovered, which may provide a protective mechanism for cardiovascular disease [21]. Here, through a series of purification steps, we acquired an ACE inhibitory protein LTP from the wild edible mushroom *L. tricolor*, which may be beneficial to hypertensive patients.

**Table 2:** N-terminal sequences of ACE inhibitory protein from *L. tricolor* and ACE inhibitory peptides from other mushrooms.

Mushroom Species	Peptide/Protein	Mode of Inhibition of ACE	Mol Weight (Da)	IC_50_
*Leucopaxillus tricolor*	(N-terminal) DGPTMHRQAVADFKQ	competitive	86,000	1.64 mg∙mL^−1^
*Agaricus bisporus* [12]	AHEPVK RIGLF PSSNK	competitive competitive non-competitive	605.3 679.5 532.3	63 μM 116 μM 129 μM
*Tricholoma giganteum* [16]	GQP	competitive	301.1	0.04 mg∙mL^−1^
*P.cystidiosus* [22]	AHEPVK GPSMR	competitive	679.5 546.3	62.8 μM 277.5 μM
*Hypsizygus marmoreus* [23]	LSMGSASLSP	non-competitive	567.3	0.19 mg∙mL^−1^
*G. lucidum* [20]	Cystathionine beta synthase-like protein,	ND	49,000	<0.2 mg∙mL^−1^
	DEAD/DEAH box helicase-like protein,		43,700	
	Paxillin-like protein,		46,800	
	Alpha/beta hydrolase-like protein		37,100	
*P.cornucopiae* [24]	RLPSEFDLSAFLRA RLSGQTIEVTSEYLFRH	competitive non-competitive	1622.8 2037.2	0.46 mg∙mL^−1^ 1.14 mg∙mL^−1^
*Pholiota adiposa* [25]	GQGGP	ND	414.0	0.044 mg∙mL^−1^
*Grifola frondosa* [14]	VIQKYP	competitive	ND	0.097 mg∙mL^−1^

“ND” means “Not Detected”.

Table 2 lists the ACE inhibitory proteins and peptides from various edible mushrooms. Peptides from *Tricholoma giganteum* and *Pholiota adiposa* exhibited ACE inhibitory activity with the lowest IC_50_ values which were 0.040 mg∙mL^−1^ and 0.044 mg∙mL^−1^, respectively. The ACE inhibitory protein from *L. tricolor* manifested ACE inhibitory activity with higher IC_50_ value (1.64 mg∙mL^−1^) among the listed mushroom ACE inhibitors. It was previously mentioned that the IC_50_ values of ACE inhibitory activity of two ACE inhibitory proteins from sweet potato storage root were 151.8 mg∙mL^−1^ and 187.96 mg∙mL^−1^, respectively [18,19]. We can see that the ACE inhibitory protein from *L. tricolor* had a higher ACE IC_50_ value than peptides, which may be attributed to its large size and increased difficulty to access the active site of ACE than peptides.

The similarity of this study and the study on peptide from fertilized eggs [26] is the purification characteristic, both use ion-exchange chromatography and FPLC, without using RP-HPLC. Most of the purification methods for ACE inhibitors included HPLC (Table 2), like that on peptide from walnut protein [27] and so on. RP-HPLC is the predominant technique used in the purification of peptides with antihypertensive activity [28]. Among Superdex columns, the Peptide 10/300 GL column and Peptide HR 10/30 column are mostly chosen to purify compounds with M.W.s ranging from 100 to 7000 [29]. Because the LTP is a protein, the Superdex 75 HR column was employed.

### 2.3. Identification of the ACE Inhibitor

The finding of a single band in SDS-PAGE for purified LTP, coupled with the results of Superdex 75 chromatography, led us to conclude that the ACE inhibitor LTP was a homodimer with a molecular mass of 86 kDa (Figure 2b) within the molecular mass range of endogenous ACE inhibitors which runs from 50 to100 kDa [21].

The N-terminal amino acids sequence of LTP was DGPTMHRQAVADFKQ and was compared with those protein and peptides of other reported edible mushrooms in Table 2. According to the report of Wu *et al*., for tripeptides, the most favorable residues for the carboxyl terminus are aromatic amino acids, while the intermediate are the positively charged amino acids, and hydrophobic amino acids are preferred for the N-terminus [30]. This is in line with the findings of Lau and coworkers [22] which show that the hydrophobicity of amino acids have great influence on ACE inhibitory activity. The percentage of hydrophobicity of the N-terminal amino acids of ACE inhibitor protein LTP was 40%. The high percentage of hydrophobicity was in agreement with the characteristic of ACE inhibitor.

**Table 3:** Internal amino acid sequences of ACE inhibitor from *L. tricolor* identified by LC-MS/MS.

Identified Peptide	Percentage of Hydrophobic Amino Acids (%)	Mass	*m*/*z*	Expected Value
VLITTDLLAR	60.0	1113.6732	557.8439	0.047
LAVNMVPFPR	80.0	1142.624	572.3193	0.06
ALLFGISGLR	60.0	1045.6632	523.8389	0.13
VAPEEHPVLLTEAPLNPK	61.1	1953.0543	652.0254	5.6
AVGKVLPALAGK	66.7	1122.6681	562.3413	7.7
FELTGIPPAPPR	66.7	1293.6608	432.2275	22
AAGGVAALLK	70.0	869.5336	435.7741	24

Hydrophobic amino acid residues are underlined.

Several internal sequences of the purified ACE inhibitory protein LTP were elucidated by LC-MS/MS using a LTQ-Orbitrap mass spectrometer. As shown in Table 3, there were seven peptides containing more hydrophobic amino acid residues and all the N-terminal amino acids were hydrophobic residues, which was in accordance with the characteristic of the potential ACE inhibitor. The ACE inhibitory protein LTP contained these peptides and the N-terminal amino acids were also in agreement with the characteristic of ACE inhibitor, hence we can conclude that the purified LTP indeed was an ACE inhibitor and deserves further study.

### 2.4. Mode of Inhibition of ACE Inhibitor

**Figure 3:**
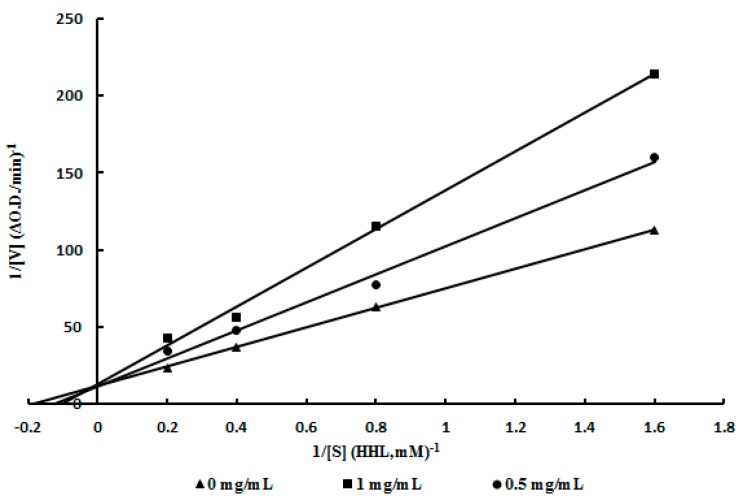
The pattern of LTP to ACE. Lineweaver-Burk plot of ACE inhibitor LTP: (▲) control, (●) 0.5 mg LTP/mL, and (■) 1 mg LTP/mL. The intersection of the three lines on the vertical axis signified that the purified ACE inhibitor LTP was a competitive inhibitor.

A Lineweaver-Burk plot was used to analyze the inhibition pattern of the purified ACE inhibitor LTP. As shown in Figure 3, with the increase of the concentration of LTP (from 0 to 1 mg∙mL^−1^), three straight lines intersected at the same point on the 1/*v* axis of the Lineweaver-Burk plot, indicating the same maximum velocity regardless of inhibitor concentration, whereas the slopes of these straight lines differed. Thus it was concluded that the purified ACE inhibitor LTP was a competitive inhibitor. Competitive ACE inhibitors purified from edible mushrooms, such as *A. bisporus*, *T. giganteum*, *P. cystidiosus*, *P. cornucopiae* and *G. frondosa* have been reported (Table 2). Noncompetitive inhibitors are found in *A. bisporus*, *Hypsizygus marmoreus* and *P. cornucopiae*. From Table 2, it can see that both competitive and non-competitive ACE inhibitors were found in *A. bisporus* and *P. cornucopiae* [12,14,16,22,23,24]. There were also competitive ACE inhibitors isolated from other food sources, for example, hen’s eggs and marine shrimps [31,32]. Unfortunately, the relationship between the mode of inhibition and structure of these peptides has not been elucidated [33].

### 2.5. Antihypertensive Action of the Purified ACE Inhibitor

**Figure 4:**
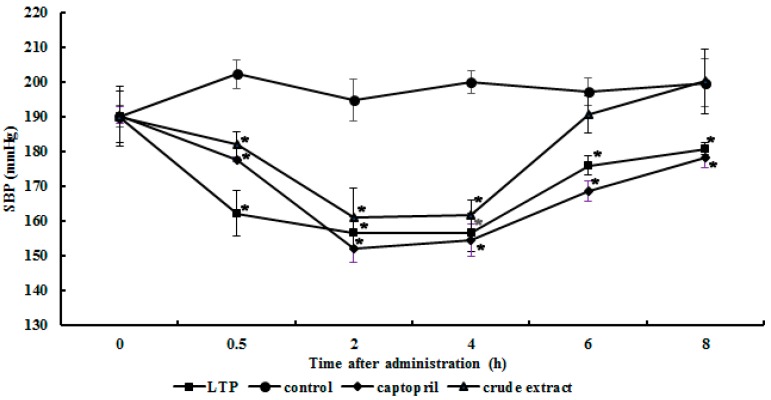
Changes in systolic blood pressure (SBP) of spontaneously hypertensive rats after oral administration of purified ACE inhibitor LTP and water extract of *L. tricolor*. Single oral administrations were performed with a dosage of 100 mg LTP/kg body weight and 200 mg water extract/kg body weight, respectively. SBP was measured 0, 0.5, 2, 4, 6 and 8 h after administration. (*) Significantly different from control at *p* < 0.05 by student’s *t*-test. (●), 0.9% saline solution; (◆), captopril; (■), ACE inhibitor LTP; (▲), water extract of *L. tricolor*.

In the single-dose and short-time test, the antihypertensive activity of LTP was evaluated by measuring the SBP in SHRs at 0.5, 2, 4, 6 and 8 h after oral administration as shown in Figure 4. Half an hour after administration of captopril (100 mg∙kg^−1^ BW), LTP (100 mg∙kg^−1^ BW) and the crude extract of *L. tricolor* (200 mg∙kg^−1^ BW), the blood pressure of all three groups had undergone a slight decrease. The maximum decline exhibited by the three groups was observed at 2 h, and the decrements in SBP were 38, 34 and 43 mmHg, respectively. Subsequently the blood pressure of all groups increased gradually. The dosage of crude extract of *L. tricolor* was 200 mg∙kg^−1^, which corresponded to 0.58 g of dried *L. tricolor*. This may provide evidence for *L. tricolor* being a functional food. Furthermore, no allergic reactions or coughing was observed on the day of the experiment and on the following day.

There have been many reports on the antihypertensive effect of edible mushrooms and other foodstuff on SHRs. The water extract of *H. marmoreus* at the dosage of 800 mg∙kg^−1^ brought about a 14.4% decrease in blood pressure in SHRs from 180 mmHg to 154 mmHg [23]. In the present study, the water extract of *L. tricolor* (200 mg∙kg^−1^) lowered the blood pressure by nearly 22.6%, but the reduction was less than that effected by *P. cornucopiae* (27.8%) [24]. Other components from mushrooms had also been investigated, for instance, in a single-dose test, the protein fraction, hot water extract and polysaccharide fraction from *P*. *nebrodensis* decreased SBP in SHRs. In a continuous-dose, the dry power and non-dialyzable fraction from *P*. *nebrodensis* also showed suppression of increase in blood pressure [34]. This may explain why the crude extract of *L. tricolor* had similar effect to the purified ACE inhibitor LTP in lowering the blood pressure.

## 3. Experimental Section

### 3.1. Materials

Fruiting bodies of *Leucopaxillus tricolor* were collected from Hailaer (in Mongolia Province) and identified by ITS. *Grifola frondosa*, *Gloeostereum incarnatum* and *Oudemansiella radicata* were purchased from a local market in Beijing. *Russula aeruginea*, *Morchella vulgaris*, *Ramaria botrytoides*, *Boletus bicolor*, *Tuber micheli*, and *Boletus edulis* were collected from Yunnan Province in China. Phenylmethylsulfonyl fluoride (PMSF), rabbit lung ACE, and hippuryl-L-histidyl-L-leucine (HHL) were purchased from Sigma-Aldrich (St. Louis, MO, USA). All solvents and chemicals used in this study were of analytical and HPLC grade. Twelve-week-old male spontaneously hypertensive rats (SHRs) were purchased from Beijing Vital River Experimental Animal Technical Co., Ltd. (Beijing, China).

### 3.2. Purification of ACE Inhibitor

Dried fruiting bodies (200 g) of *L. tricolor* were blended in distilled water (1:20, w:v) at 4 °C overnight. The mixture was centrifuged at 9000 rpm for 20 min at 4 °C, and the supernatant was subjected to ultrafiltration with a 10,000 M.W. cut-off membrane (Labscal TFF System, Millipore Co., Billerica, MA, USA).The filtrate and solution of the filter-cake were assayed for ACE inhibitory activity as described below. The active fraction was loaded on a Q-Sepharose column (3 cm × 25 cm, GE Healthcare, Uppsala, Sweden) which had previously been equilibrated with 10 mM Tris-HCl buffer (pH 7.5), and then eluted with a linear gradient of 0–1 M NaCl in the same buffer. After dialysis, the fraction with ACE inhibitory activity was chromatographed on another Q-Sepharose column (3 cm × 15 cm), and eluted with a 0–1 M NaCl concentration gradient in 10 mM Tris-HCl buffer (pH 7.2). Following dialysis, the active fraction was further purified by fast protein liquid chromatography (FPLC) on a gel filtration Superdex 75 10/300 GL column (GE Healthcare, Uppsala, Sweden) in 10 mM Tris-HCl buffer (pH 7.5) using an AKTA Purifier (GE Healthcare, Uppsala, Sweden).

### 3.3. Assay of ACE Inhibitory Activity

ACE was prepared following the protocol described by Hayakari *et al*. [35] with some modifications. Briefly, a fresh rabbit lung (5 g) without connective tissues was washed with pre-cooled NaCl solution (0.15 M), cut into pieces, and then homogenized in 45 mL ice-cold 0.1 M sodium borate buffer, pH 8.3, containing 0.25 M sucrose and 0.1 mM PMSF. The homogenate was dialyzed overnight in 0.1 M sodium borate buffer, pH 8.3 at 4 °C. The mixture was then centrifuged at 9000 rpm for 40 min at 4 °C, and the resulting supernatant was centrifuged at 9000 rpm for 40 min at 4 °C. The supernatant was aliquoted and stored at −20 °C for further experiments.

ACE inhibitory activity was assayed as described by Cushman *et al*. [36] with modifications. The reaction mixture containing 20 μL ACE from rabbit lungs and 20 μL inhibitor was pre-incubated for 5 min at 37 °C, and then 150 μL of 5 mM HHL (0.1 M, sodium borate buffer pH 8.3 and 0.3 M NaCl) was added to start the reaction. The reaction was terminated by the addition of 500 μL of 1 M HCl after incubation for 30 min. The liberated hippuric acid was extracted with 1.2 mL of ethyl acetate, and 0.8 mL of the extract was heat-evaporated at 95 °C for 35 min to remove the ethyl acetate. The residue was then dissolved in 0.8 mL distilled water and measured spectrophotometrically at 228 nm to estimate ACE inhibitory activity. The activity was calculated using the following formula:

$$\text{Inhibition activity }(\text{\%})=(1-\frac{A-B}{C-D})\times 100$$

where A is the absorbance of ACE + distilled water, B is the absorbance of distilled water, C is the absorbance of ACE + inhibitor, and D is the absorbance of inhibitor + water. The concentration of the inhibitor required to inhibit 50% of the ACE activity under the above assay conditions was defined as IC_50_.

### 3.4. Molecular Mass Determination and Amino Acid Sequence Analysis of the Isolated ACE Inhibitor

The molecular mass of the purified ACE inhibitor was determined by sodium dodecyl sulfate-polyacrylamide gel electrophoresis (SDS-PAGE) and gel filtration using an FPLC system. SDS-PAGE was conducted in accordance with the procedure of Laemmli and Favre, using a 15% resolving gel and a 5% stacking gel [37]. Gel filtration was carried out using a Superdex 75 10/300 GL column, which had been calibrated with molecular mass standards.

The N-terminal amino acid sequence of the inhibitor was analyzed by automated Edman degradation using a Perkin Elmer protein sequencer (Model 491, Applied Biosystems, Foster, CA, USA). Identification of the peptide sequences of the purified inhibitor was performed as follows: the inhibitor was digested with trypsin and then dissolved in 0.1% formic acid and 2% acetonitrile for liquid chromatography-tandem mass spectrometry (LC-MS/MS) analysis using a LTQ-Orbitrap mass spectrometer (Thermo Electron, Bremen, Germany).

### 3.5. Determination of Mode of Inhibition of ACE

To determine the type of inhibition of ACE by the purified inhibitor, various concentrations (0.63, 1.25, 2.5 and 5 mM) of HHL were incubated with ACE in the absence and presence of the inhibitor at two different concentrations (1 mg∙mL^−1^ and 0.05 mg∙mL^−1^). The ACE inhibitory activity was determined following the aforementioned method. From the Lineweaver-Burk plot, the kinetic parameters V_max_ and K_m_ in the absence and presence of the inhibitor were determined.

### 3.6. Antihypertensive Action in Spontaneously Hypertensive Rats (SHRs)

All experiments were performed in accordance with the Regulations of Experimental Animal Administration issued by the State Committee of Science and Technology of the People’s Republic of China and with the approval of the Animal Experimentation Ethics Committee of The Chinese University of Hong Kong which had been procured prior to the animal experiments. The SHRs were fed acclimatized for a week, housed in cages and maintained in an air-conditioned room (25 ± 1 °C) and kept on a 12:12 light-dark cycle. Food and tap water were provided *ad libitum*. During the acclimatization period prior to the test, the blood pressures of the rats were measured four times weekly.

The rats were divided into four groups, with each group containing four rats: 0.9% saline solution was administered orally to the blank control group. The inhibitor group received 100 mg inhibitor /kg body weight, and the crude water extract group received 200 mg crude water extract of *L. tricolor* /kg body weight. The inhibitor was dissolved in the same volume of saline solution for administration. The systolic blood pressure (SBP) was measured by the tail-cuff method with a programmable BP-100A electro-sphygmomanometer (Chengdu Taimeng Software Co., Ltd., Chengdu, China) before drug/sample administration, and again 0.5, 2, 4, 6 and 8 h after drug/sample administration.
